# Elevated lncRNA MIAT in peripheral blood mononuclear cells contributes to post-menopausal osteoporosis

**DOI:** 10.18632/aging.204001

**Published:** 2022-04-05

**Authors:** Rui Li, Ting-Ting Shi, Qiang Wang, Yong-Xian Zhang

**Affiliations:** 1 Department of Traumatic Orthopedic Surgery, PLA 960th Hospital, Ji’nan 250031, China; 2 Oncology Department, PLA 960th Hospital, Ji’nan 250031, China

**Keywords:** post-menopausal osteoporosis, lncRNA MIAT, peripheral blood mononuclear cells, miR-216a, p38MAPK

## Abstract

Inflammatory cytokines contribute to the development of osteoporosis with sophisticated mechanisms. Globally alteration of long-chain non-coding RNA was screened in osteoporosis, while we still know little about their functional role in the inflammatory cytokine secretion. In this study, we collected the peripheral blood mononuclear cells (PBMCs) from post-menopausal osteoporosis patients to measure lncRNA MIAT (lncMIAT) expression levels, and explored the molecular mechanism of lncMIAT induced inflammatory cytokine secretion. We identified increased lncMIAT expression in the PBMCs of post-menopausal osteoporosis patients, which was an important predictive biomarker for the diagnosis. LncMIAT expression in PBMCs was positively correlated with the inflammatory cytokine secretion. Mechanism study indicated that lncMIAT increased the expression levels of p38MAPK by crosstalk with miR-216a in PBMCs. The lncMIAT/miR-216a/p38MAPK signaling contributed predominantly to the increased inflammatory cytokine secretion in the PBMCs from postmenopausal osteoporosis. In conclusion, we identified that increased lncMIAT in PBMCs induced inflammatory cytokine secretion, which contributed to the development of post-menopausal osteoporosis. lncMIAT/miR-216a axis was critical for the regulation of AMPK/p38MAPK signaling, which may be a promising therapeutic target for osteoporosis treatment by inflammatory cytokine inhibition.

## INTRODUCTION

Elevated incidence of osteoporosis, which is described as an imbalance in bone formation and resorption, is observed in the elderly, especially the post-menopausal women. Post-menopausal osteoporosis progression induces bone remodeling, which will lead to bone fragility and fracture [1]. Previous investigation was focused on the molecular biological alterations of osteoclasts and osteoblasts [2]. However, we have not achieved satisfactory therapeutic effects with hormone replacement or calcium supplement [3], because of the poisonous side effect, malabsorption, and other reasons [4]. Therefore, expanded investigating fields related to post-menopausal osteoporosis are critical for the therapeutic improvement.

Adenosine monophosphate-activated protein kinase (AMPK) and downstream mitogen-activated protein kinase (MAPK) play a crucial role for the inflammatory cytokine secretion [5]. Of note, p38MAPK signaling plays a dominant role in the inflammatory cytokine secretion of peripheral blood mononuclear cells (PBMCs) [5, 6]. Recent studies supported inflammatory cytokines contributed to the communication between immune system and bones [7]. For instance, tumor necrosis factor-alpha (TNF-α) and interleukin-6 (IL-6) regulate osteoblasts activity to alter bone metabolism [8–10], which are correlated to immune-mediated osteoporosis [11]. Bone resorption is predominantly induced by IL-6, IL-1β and TNF-α in osteoporosis, especially in the post-menopausal patients [12, 13]. In this regard, the functional role of AMPK/MAPK signaling in the pathological process of post-menopausal osteoporosis needs further clarification.

Globally alteration of long-chain non-coding RNAs (lncRNAs) were screened in osteoporosis [14], which supported their biological roles in the disease progression. Systematic analysis of peripheral blood lymphocytes indicated a series of differentially expressed lncRNAs in the patients with postmenopausal osteoporosis [15]. Among them, lncRNA MIAT (myocardial infarction-associated transcript, lncMIAT) showed significant upregulation in the PBMCs of osteoporosis patients [15]. lncMIAT is located on chromosome 22q12.1, which shows low expression in human adipose-derived stem cells during osteogenic differentiation [16]. lncMIAT knockdown enhanced osteogenic differentiation of human adipose-derived stem cells [16]. The studies on immune system regulation by lncRNAs in osteoporosis progression are still rare, especially for the PBMCs [17]. Further studies are still needed to explore its functional role in post-menopausal osteoporosis.

In this study, we measured the expression status of lncMIAT in the PBMCs, and investigated the regulation mechanism of inflammatory cytokine secretion in post-menopausal osteoporosis. Our results further interpreted the correlation of inflammatory cytokines with the pathogenesis of post-menopausal osteoporosis.

## RESULTS

### Increased lncMIAT expression in the PBMCs from post-menopausal osteoporosis patients

**Figure 1:**
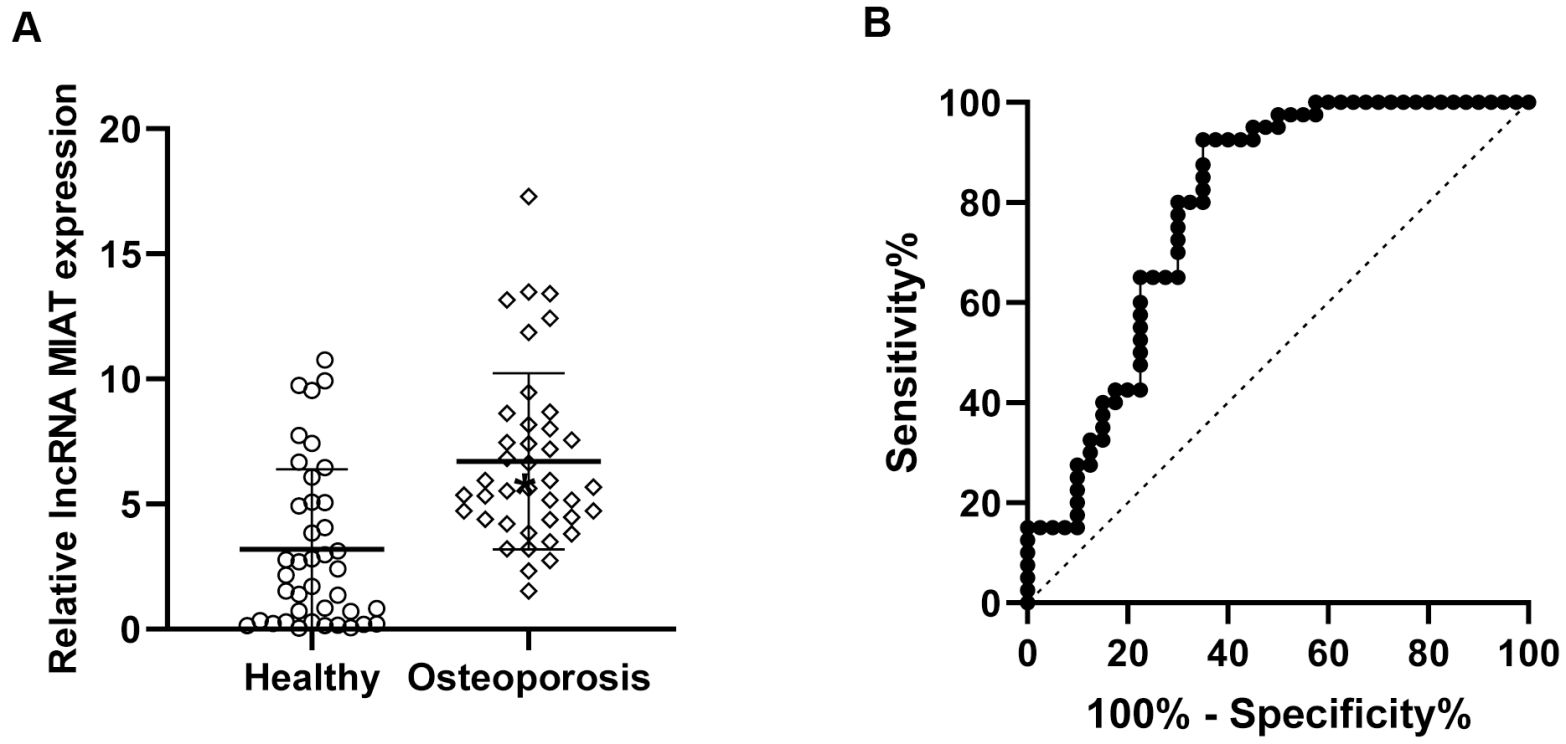
**Increased lncMIAT levels in PBMCs from post-menopausal osteoporosis patients.** (**A**) Compared to the 40 healthy participants (Healthy group), lncMIAT showed increased levels in 40 post-menopausal osteoporosis patients (Osteoporosis group). p < 0.05. (**B**) The diagnostic value of lncMIAT expression in PBMCs was analyzed by ROC curve. Aera under the curve was 0.788, Cutoff value was 3.168, 95% confidence interval was 0.69-0.89 and p < 0.001.

We firstly analyzed the lncMIAT expression in PBMCs from post-menopausal osteoporosis patients and corresponding healthy participants. Reverse transcription-quantitative PCR (RT-qPCR) assays were performed for the expression levels of lncMIAT. The results indicated higher lncMIAT levels in osteoporosis patients than the healthy participants (Figure 1A, p < 0.05). Further ROC analysis was performed for the diagnostic values of lncMIAT in osteoporosis. Our results showed that the area under the curve was 0.788, with a standard error of 0.052 and a 95% confidence interval of 0.69-0.89 (Figure 1B, p < 0.001). The results supported that the increased lncMIAT expression in PBMCs distinguished post-menopausal osteoporosis patients from healthy participants, which was a promising biomarker for the diagnosis of osteoporosis.

### lncMIAT increases the inflammatory cytokine secretion of PBMCs

**Figure 2:**
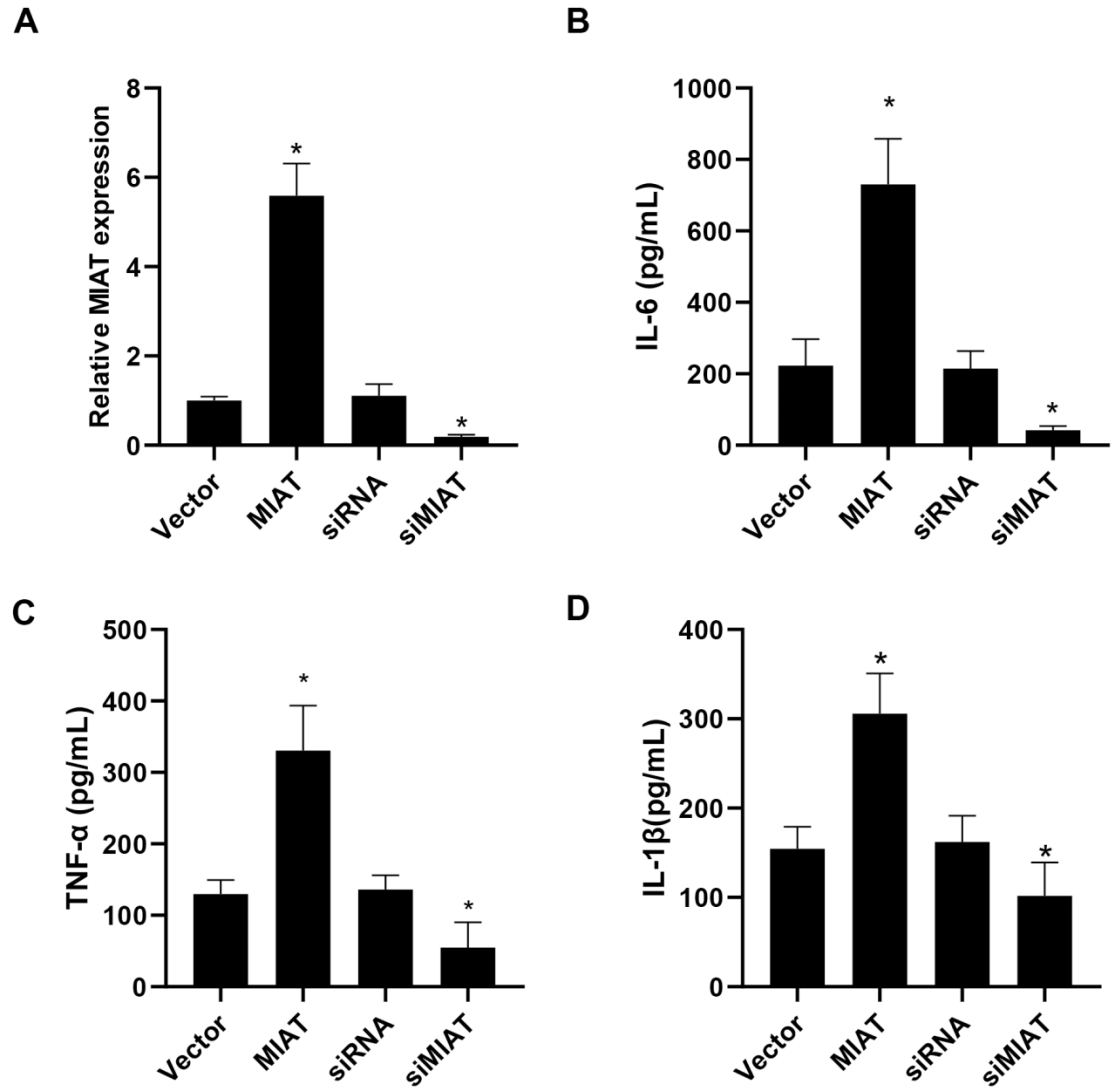
**lncMIAT upregulates inflammatory cytokine secretion in PBMCs.** (**A**) Transfection efficacy of lncMIAT and siMIAT was measured in PBMCs by RT-qPCR assays. GAPDH was used as control. (**B**–**D**) ELISA assays of IL-6, TNF-α and IL-1β were performed with the cultured medium of transfected cells. Elevated lncMIAT expression increased inflammatory cytokine secretion in PBMCs, while lncMIAT silencing reduced inflammatory cytokine levels. *, p < 0.05.

Then we investigated the correlation of inflammatory cytokine levels and lncMIAT expression in the PBMCs. Firstly, lncMIAT over-expression or silencing cells were established with the PBMCs. The expression levels of lncMIAT were confirmed with RT-qPCR assays (Figure 2A). Then ELISA assays were performed for the levels of inflammatory cytokine in the medium of cultured cells. Exogenous lncMIAT over-expression in PBMCs significantly increased the levels of IL-6, while lncMIAT silencing decreased the secretion of IL-6 (Figure 2B, p < 0.05). Similar results were also observed in TNF-α and IL-1β (Figure 2C, 2D, p < 0.05). The results supported that lncMIAT was participated in the secretion of inflammatory cytokines in PBMCs from post-menopausal osteoporosis patients.

### lncMIAT increases p38MAPK expression to promote inflammatory cytokine secretion

**Figure 3:**
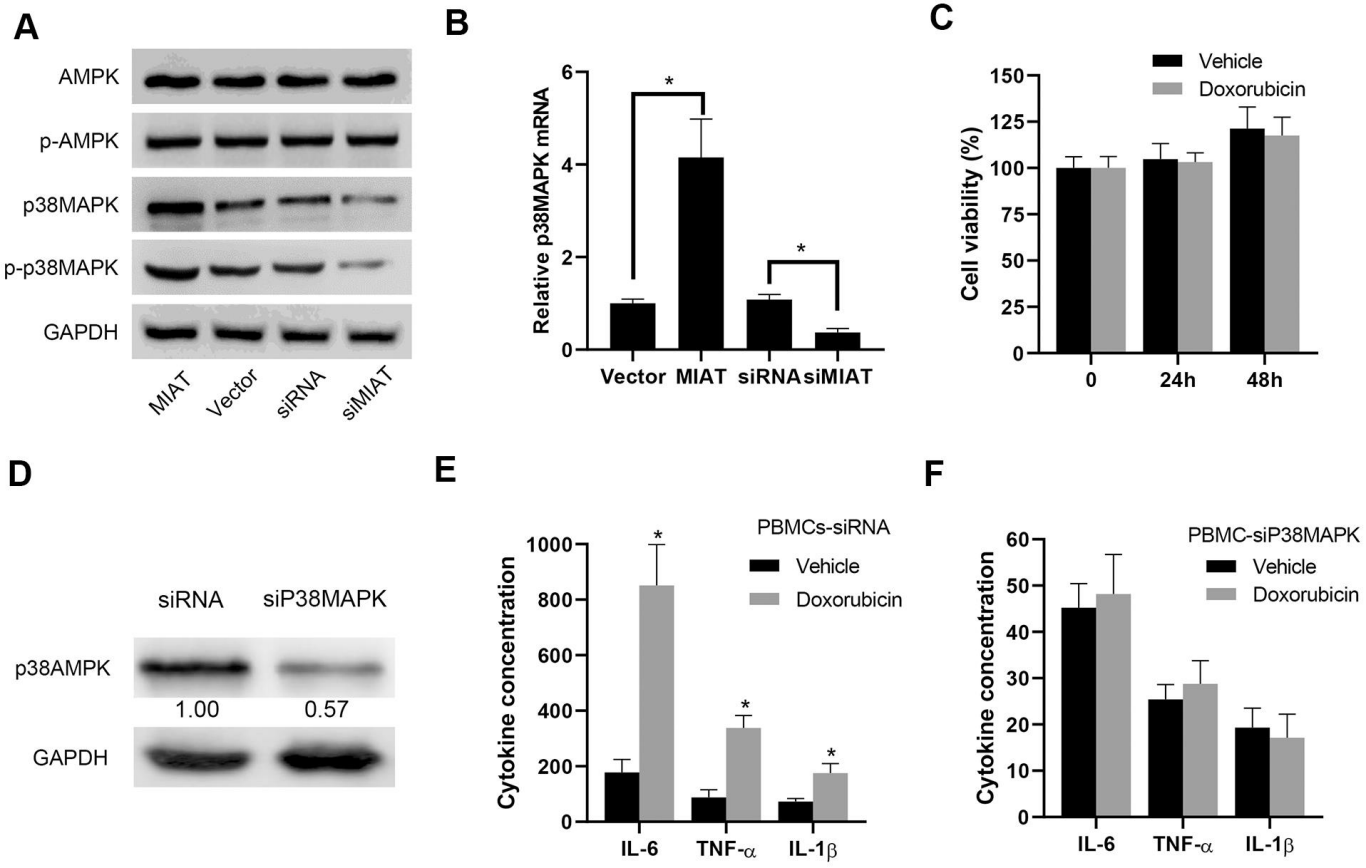
**lncMIAT increases p38MAPK expression to upregulate inflammatory cytokine secretion.** (**A**) The expression levels of AMPK, p38MAPK and corresponding phosphorylated proteins were measured in the lncMIAT overexpressing and silencing PBMCs with Western blot assays. GAPDH was used as loading control. (**B**) The mRNA expression of p38MAPK was analyzed with the transfected PBMCs and corresponding control cells with PT-qPCR assays. (**C**) CCK-8 assays were performed to evaluate the PBMCs cell viability, which were treated with Doxorubicin (1 μM) for 24h or 48h. (**D**) p38MAPK knockdown PBMCs were established. The p38MAPK protein levels were confirmed with Western blot assays. GAPDH was used as control. (**E**, **F**) The inflammatory cytokine secretion of IL-6, TNF-α and IL-1β were measured with ELISA assays in the transfected PBMCs, which were treated with Doxorubicin (1 μM). *, p < 0.05.

AMPK/p38MAPK signaling pathway played a crucial role in regulating the secretion of inflammatory cytokines in PBMCs, especially lymphocytes and monocytes [5, 6]. Based on the results above, we performed further Western blot assays to measure the expression and phosphorylation status of AMPK/p38 MAPK pathway in the infected PBMCs. We observed that lncMIAT overexpression induced a dramatical upregulation of total protein expression and phosphorylation of p38MAPK in the PBMCs (Figure 3A). However, the protein level and phosphorylation of AMPK showed no significant change (Figure 3A). Moreover, the PT-qPCR analysis showed similar results of p38MAPK expression in the transfected cells (Figure 3B). We used 1 μM Doxorubicin (AMPK signal pathway inhibitor) to treat the PBMCs. The cytotoxic effects of Doxorubicin treatment indicated no significant influence in PBMCs viability (Figure 3C). Then we performed further analysis with p38AMPK knockdown, which was confirmed with Western blot assays (Figure 3D). ELISA assays indicated that Doxorubicin could significantly increase the expression of IL-6, TNF-α and IL-1β in the control cells (Figure 3E), while p38MAPK knockdown attenuated AMPK inhibition induced inflammatory cytokine secretion to a large extent (Figure 3F). Based on these results, our results suggest that lncMIAT effectively increases inflammatory cytokine secretion by upregulating p38MAPK expression.

### lncMIAT directly binds with miR-216a in PBMCs

**Figure 4:**
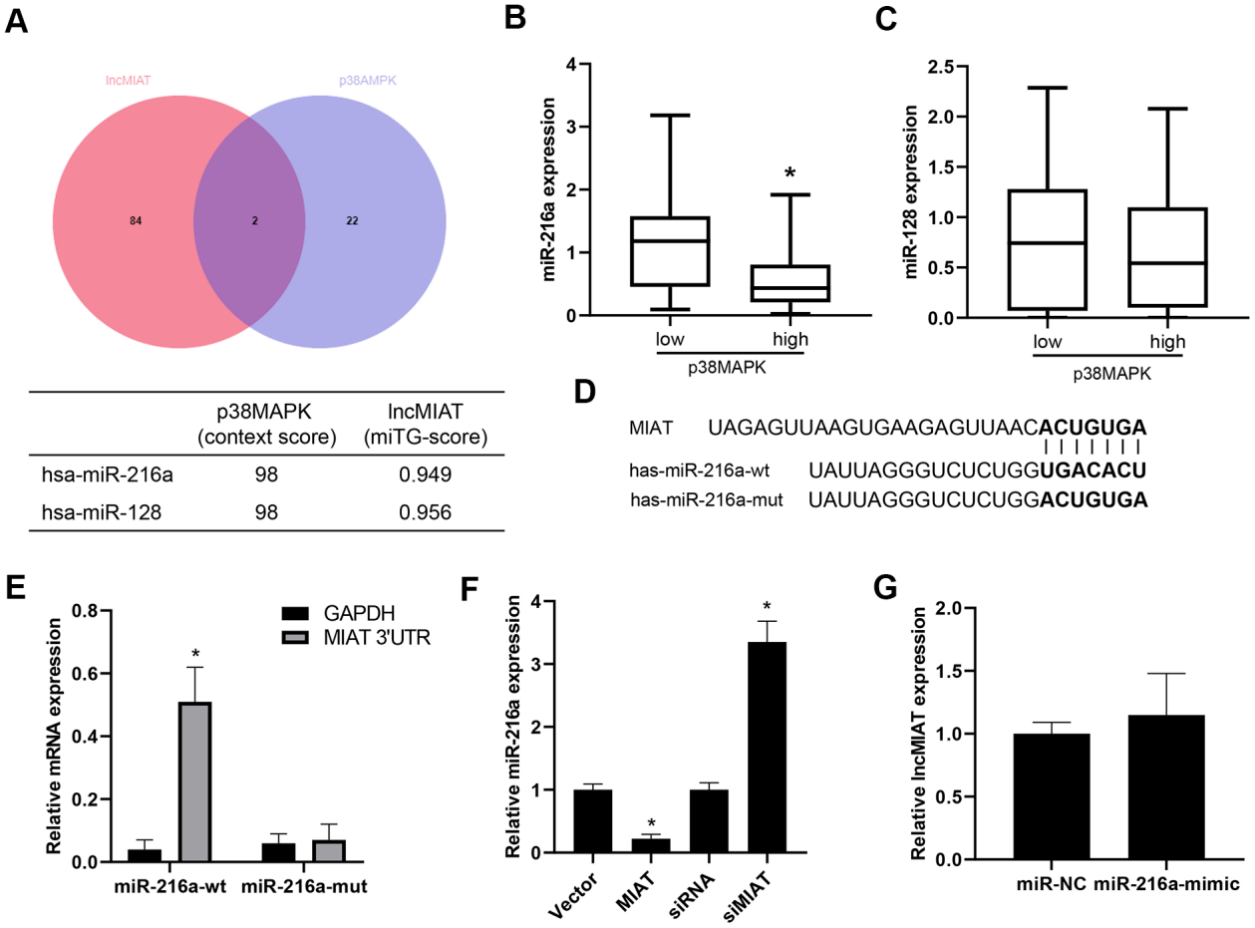
**lncMIAT directly binds with miR-216a.** (**A**) Bioinformatic analysis was performed for the miRNAs which were potentially interacted with lncMIAT and p38MAPK. VENN analysis was performed for the miRNA targets in common. (**B**, **C**) The expression levels of miR-216a and miR-128 was compared between p38MAPK high and low group in our cohort (n = 40). (**D**) LncBase v.2 analysis predicted the binding site of lncMIAT and miR-216a. Wild type and mutant miR-216a were prepared as the sequence shown in diagram. (**E**) PBMCs were co-infected with lncMIAT and miR-216a-wt or miR-216a-mut. Relative levels of lncMIAT and GAPDH were analyzed in the miR-216a pulled down pellet with RT-qPCR assays. (**F**) RT-qPCR assays were performed for the miR-216a expression in lncMIAT overexpressing or silencing cells. Negative correlation was observed between lncMIAT and miR-216a expression. (**G**) lncMIAT expression levels were detected in miR-216a negative control (NC) or mimics infected cells, which were analyzed with RT-qPCR assays. *, p < 0.05.

Further analysis was performed for the regulation mechanism of lncMIAT in p38MAPK expression. VENN analysis was performed for the potential miRNA target for lncMIAT and p38MAPK crosstalk, which screened out two promising miRNAs, miR-216a and miR-128 (Figure 4A). Further analysis was performed for the correlation of miRNAs and p38MAPK expression in PBMCs from osteoporosis patients. We observed a reverse correlation of miR-216a and p38MAPK expression, rather than miR-128 (Figure 4B, 4C). Prediction analysis with LncBase v.2 showed a promising binding site between lncMIAT and miR-216a (Figure 4D). The RT-qPCR analysis detected a higher level of lncMIAT in the wild type miR-216a pulled down pellet than the mutant ones in the infected PBMCs (Figure 4E). Moreover, RT-qPCR assays were also performed with lncMIAT overexpressing or silencing cells, which showed decreased miR-216a expression in exogenous lncMIAT expressing PBMCs, while upregulated in lncMIAT silencing cells (Figure 4F). However, miR-216a mimics transfection showed no significant impact for the expression levels of lncMIAT in PBMCs (Figure 4G). These results supported the interaction between lncMIAT and miR-216a, rather than transcriptional regulation.

### lncMIAT/miR-216a axis regulates p38MAPK expression in PBMCs

**Figure 5:**
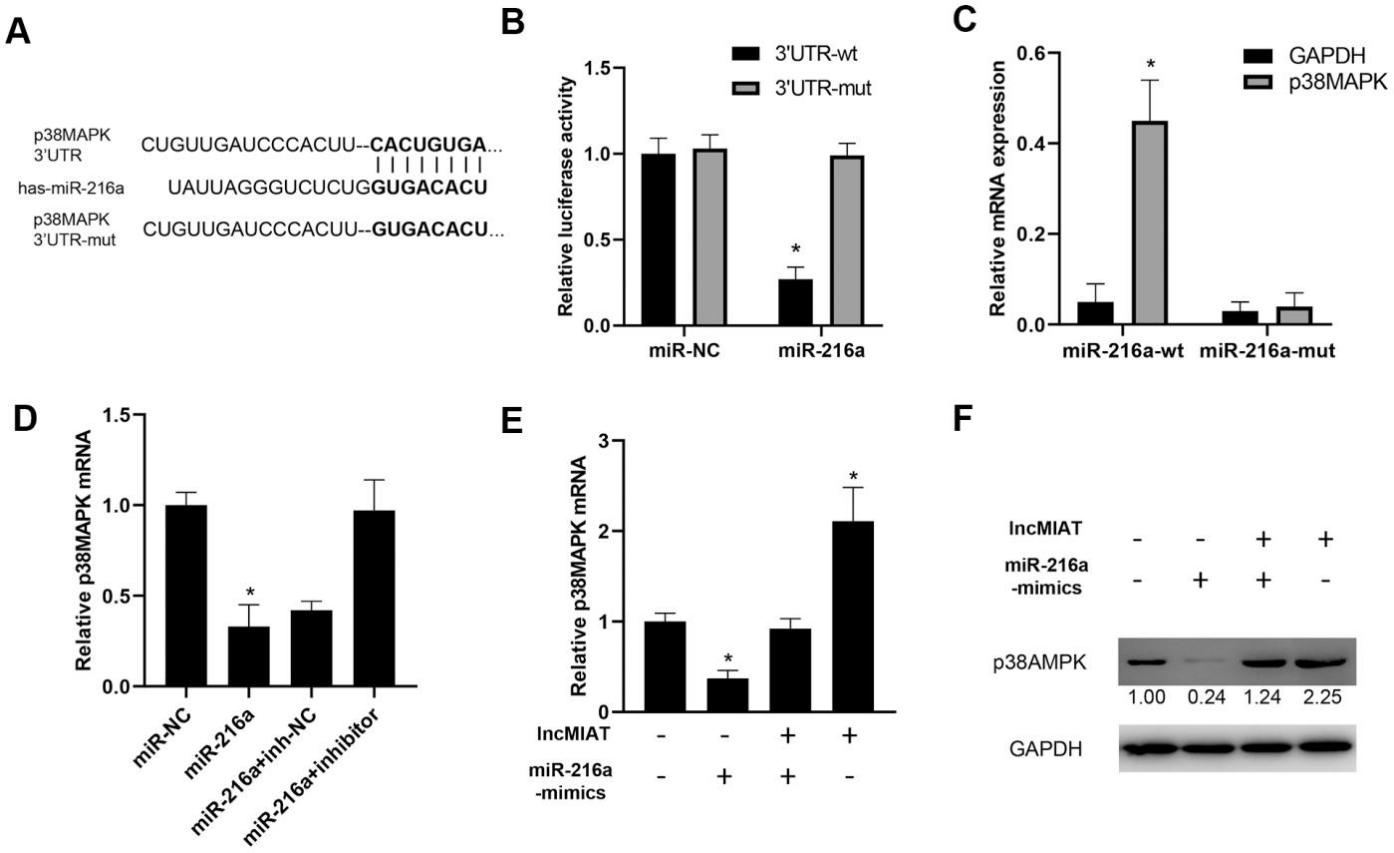
**lncMIAT/miR-216a axis regulates p38MAPK expression in PBMCs.** (**A**) Targetscan 7.2 predicted a binding site of 3’UTR of p38MAPK by miR-216a. And mutant 3’UTR of p38MAPK was prepared for further analysis. (**B**) Luciferase assays were performed with wild type or mutant 3’-UTR of p38MAPK, to examine the impact of miR-216a for luciferase activity. (**C**) Cells were transfected with wild-type or mutant miR-216a and 3’-UTR of p38MAPK. RNA pull-down assays were performed for the interacted 3’-UTR of p38MAPK. (**D**) RT-qPCR assays were performed for p38MAPK expression levels in miR-216a or inhibitor transfected cells. (**E**, **F**) lncMIAT and miR-216a mimics were co-transfected in PBMCs as indicated. The expression levels of p38MAPK mRNA were detected by RT-qPCR (**E**) and Western blot (**F**). *, p < 0.05.

We further investigated the function of lncMIAT/miR-216a on p38MAPK expression in PBMCs. Bioinformatic prediction indicated a promising binding site between the 3’ UTR of p38MAPK and miR-216a (Figure 5A). Luciferase assays indicated that the miR-216a inhibited the luciferase activity of wild type 3’-UTR of p38MAPK, while no significant influence for the mutant type (Figure 5B). Further RNA pull-down assays indicated that higher levels of 3’-UTR of p38MAPK were observed in wild-type miR-216a than mutant miR-216a (binding site mutant) (Figure 5C). Then, we overexpressed miR-216a in PBMCs and observed the downregulation of p38MAPK expression, which was attenuated by miR-216a-inhibitor (Figure 5D). Moreover, the co-transfection of lncMIAT/miR-216a mimics was performed with PBMCs. The results showed that lncMIAT/miR-216a co-transfection attenuated lncMIAT induced p38MAPK overexpression (Figure 5E). Further Western blot analysis also supported the similar results in the co-transfected PBMCs (Figure 5F). These results indicated that lncMIAT/miR-216a axis regulated the transcriptional expression of p38MAPK.

### The relationship between the expression of lncMIAT/miR-216a and p38MAPK in PBMCs

**Figure 6:**
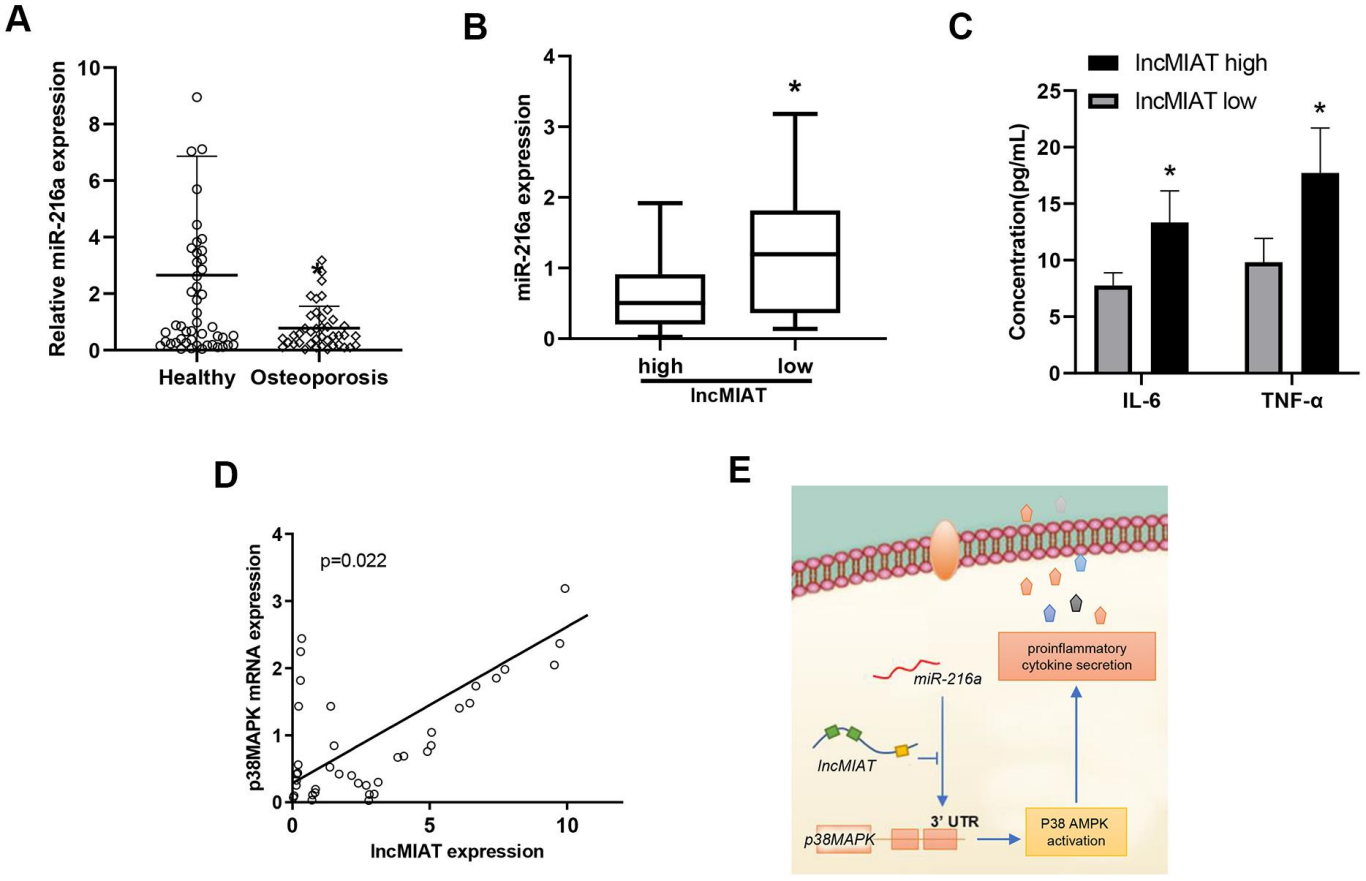
**The relationship between the expression of lncMIAT/miR-216a and p38MAPK in PBMCs.** (**A**) The comparison of miR-216a levels in PBMCs was performed between osteoporosis patients and healthy participants, which was detected by RT-qPCR analysis. (**B**) The miR-216a expression levels were compared between low lncMIAT and high lncMIAT groups. (**C**) The plasma concentration of IL-6 and TNF-α was compared between lncMIAT high and low expression group in our cohort. Totally 32 post-menopausal patients with complete test results were collected for the analysis. (**D**) Linear regression model was used for the correlation of lncMIAT and p38MAPK expression in PBMCs from osteoporosis patients. *, p < 0.05. (**E**) A graphic summary for LncMIAT contributing to proinflammatory cytokine secretion in post-menopausal osteoporosis.

Further clinical significance of miR-216a and p38MAPK was evaluated with the collected PMBCs in our cohort. RT-qPCR assays were performed for the miR-216a and p38MAPK expression levels. Our results showed lower levels of miR-216a in PBMCs from the osteoporosis patients than the healthy participants (Figure 6A). More importantly, significant lower levels of miR-216a were also observed in lncMIAT high-expressing patients than the low lncMIAT patients (Figure 6B). Furthermore, we observed higher plasma concentration of IL-6 and TNF-α in lncMIAT high post-menopausal patients than the low expression ones (Figure 6C). More importantly, a significant positive correlation was also identified for the expression levels of lncMIAT and p38MAPK in PBMCs (Figure 6D). The results support that lncMIAT/miR-216a contributes to the activation of AMPK/p38MAPK pathway in PBMCs derived from osteoporosis patients.

## DISCUSSION

Current evidence supported that lncRNAs contributed to the cellular and molecular mechanisms linked to osteoporosis [18]. Specially, lncMIAT worked as an osteogenesis inhibitor of adipose derived stem cells in an mice model of heterotopic bone formation [16]. Our present study focused on the inflammatory cytokine secretion of the PBMCs from osteoporosis patients. We identified that elevated lncMIAT induced inflammatory cytokine secretion in PBMCs, which was involved in the development of post-menopausal osteoporosis. The crosstalk between lncMIAT and miR-216a was involved in the transcription of p38MAPK, which was critical for the AMPK/MAPK signaling activation induced inflammatory cytokine secretion.

Elevated lncMIAT expression in PBMCs was identified from RNA sequence analysis in post-menopausal osteoporosis patients [15]. lncMIAT is involved in osteogenic differentiation of human adipose-derived stem cells *in vitro* and *in vivo* [16]. Molecular mechanism studies indicated that lncMIAT interacted with miR-150-5p to inhibit the osteogenic differentiation which was correlated with inflammatory cytokine and oxidative stress [19, 20]. Furthermore, lncMIAT inhibition contributes to the therapeutic effects in osteonecrosis amelioration, such as Huo Xue Tong Luo capsule [21]. In this study, we identified a positive correlation between lncMIAT and p38MAPK expression in PBMCs. Subsequent cellular studies confirmed that lncMIAT promoted the secretion of IL-6, TNF-α and IL-1β, which were important inflammatory cytokines for the disease progression of osteoporosis [7]. Given lncMIAT serves as an inhibitor for AMPK signaling activation, the crosstalk between PBMCs and osteoblasts or osteoclasts is of great value to investigate the immune system in osteoporosis. Further studies will be performed to characterize the potential interactions between inflammation factors and bone metabolism.

We proved that miR-216a was involved in p38MAPK transcriptional regulation by lncMIAT. Our results indicated that miR-216a interacted with lncMIAT to regulate the cytokine secretion of PBMCs. Molecular analysis showed a direct binding of lncMIAT and miR-216a. Luciferase activity analysis indicated that miR-216a bound to 3’UTR of p38MAPK to degrade the post-transcriptional regulation. Previous studies indicated that miR-216a worked as a tumor suppressor to inhibit tumor cell proliferation, invasion and metastasis [22, 23]. MiR-216a also interacted with lncRNA DANCR to promote hepatocellular carcinoma malignancy [24]. Recent studies also indicated that miR-216a attenuated the dexamethasone induced osteogenesis suppression. The c-Cbl-mediated PI3K/AKT pathway mediated miR-216a induced the osteoblast differentiation and bone formation effects [25]. In this study, we further expended the functional role of miR-216a in post-menopausal osteoporosis. Our results supported that miR-216a was a beneficial factor for osteoporosis by inhibiting p38MAPK signaling and inflammatory cytokine secretion.

Accumulated evidence supported that IL-6, IL-1β, and TNF-α contributed to bone resorption [26]. Inflammatory cytokines enhance the osteoclast activity predominantly by regulating the RANKL/OPG balance [27]. Proinflammatory cytokines also participate in inflammatory responses, such as the activation of macrophage and antigen presentation [28]. Consistently, we observed higher cytokine levels in PBMCs of post-menopausal osteoporosis patients than the healthy controls. More importantly, elevated lncMIAT expression showed diagnostic value for post-menopausal osteoporosis. Furthermore, lncMIAT/miR-216a axis activated p38MAPK signaling in post-menopausal osteoporosis, which was also a critical pathway for osteoclast differentiation and bone absorption in the osteoporosis [29]. AMPK pathway activation by chemicals showed significant protective effects, such as Hydroxytyrosol [30], Ginsenoside Rg3 [31], and Zoledronate [29]. However, AMPK signaling activation also rescues osteoblastic cells from dexamethasone treatment [32], as well as ovariectomy-induced bone loss [33]. Therefore, AMPK/p38MAPK signaling activation by lncMIAT/miR-216a axis in PBMCs was considered as a potential therapeutic target for osteoporosis. Further clinical studies are needed for the clinical application.

In this study, the PBMCs were isolated with Ficoll-Paque density gradient centrifugation, which were mainly lymphocytes and monocytes [34]. To maintain the cell viability for cell culture and further mechanism study, we did not perform the flow cytometry analysis for further cell subpopulation sorting. Further functional role of PBMCs subpopulation investigation is still needed in the development of osteoporosis. Previous studies indicated that AMPK and downstream signaling played a crucial role in the secretion of cytokines [5]. NK-κB pathway plays a predominant role in mediating AMPK induced cytokine expression in endothelial cells [35], adipose cells [36], and macrophage [37]. Besides, MAPK is another important signaling to mediate inflammatory response. Among them, p38MAPK contributes dominantly to the inflammatory cytokine secretion in PBMCs, including macrophage and T cells [5, 6, 38]. We provided further evidence of the interaction of AMPK and p38AMPK signaling in PBMCs from post-menopausal osteoporosis patients. However, the functional role of other signaling was not excluded in lncMIAT induced osteoporosis, including NK-κB pathway. Moreover, disease progression and medical treatment also influence the inflammatory microenvironment. Further clinical analysis for the crosstalk is still needed in our future study.

In conclusion, increased lncMIAT was a predictive biomarker for post-menopausal osteoporosis. lncMIAT/miR-216a axis was critical for AMPK/p38MAPK signaling activation, which maybe a promising therapeutic target for improving osteoporosis treatment effects by inhibition of proinflammatory cytokines.

## MATERIALS AND METHODS

### Osteoporosis patients and healthy participants enrollment

Totally 40 diagnosed post-menopausal osteoporosis patients and corresponding 40 healthy participants were collected in this study. All the participants signed informed consent and the study was approved by the ethics committee of PLA 960^th^ Hospital. Post-menopausal osteoporosis was diagnosed according to Expert Consensus on the Diagnosis of Osteoporosis in Chinese Population (Version 3). The diagnose standard was set as -2.0 SD or 25% reduce of bone mineral density (BMD). All participants received no treatments in the last 3 months before venous blood collection. The healthy participants were collected for no significant differences in age and Body Mass Index (BMI) with osteoporosis patients.

### RT-qPCR assays

Five milliliters fresh venous blood was collected into sodium heparin tube and mixed well. Ficoll-Paque (Sigma-Aldrich, St. Louis, MO, USA) was used for PBMCs purification. Total RNA extraction was performed with TRIzol kit (Invitrogen, Carlsbad, CA, USA). Then cDNA reverse transcription and RT-qPCR assays were performed with PrimeScript RT Master Mix (Takara, Dalian, China). The relative levels of RNA were calculated with 2^-ΔΔCt^ method. Triple replicates were conducted for each target gene. Primers were designed as the follows: p38MAPK: For, 5’-TCAGTCCATCATTCATGCGAAA-3’, Rev, 5’-AACGTCCAACAGACCAATCAC-3’; miR-216a: For,5’- TGTCGCAAATCTCTGCAGG -3’, Rev, 5’-CAGAGCAGGGTCCGAGGTA-3’; U6: For, 5’-GCTTCGGCAGCACATATACTAA-3’, Rev, 5’-AACGCTTCACGAATTTGCGT-3’; lncMIAT: For, 5’- ATCCTCGAGACAAAGAGCCCTCTGCACTAG-3’, Rev, 5’- ATCGGATCCGAGCAAATGGAGACAAAGGAC-3’; GAPDH: For, 5’-CATCACTGCCACCCAGAAGACTG-3’, Rev,5’-ATGCCAGTGAGCTTCCCGTTCAG-3’.

### Cell culture and treatment

The peripheral blood was collected from the participants in the morning before breakfast. Next, the PBMCs were isolated with Ficoll-Paque density gradient centrifugation (GE Healthcare Biosciences), which were cultured in RPMI-1640 (10% FBS, 20 μg/mL streptomycin and 20 IU/mL penicillin). AMPK signaling was inhibited with Doxorubicin (Adriamycin) HCl (1 μM), which was purchased from Selleckchem (Houston, TX, USA).

### Cell transfection

Cell transfection was performed with Lipofectamine 2000 (Thermo Fisher Scientific, Inc., Waltham, MA, USA). The lncMIAT and empty plasmids, siMIAT [39], siP38MAPK [40], and scramble control were synthesized by GenePharma (Shanghai, China). The miR-216a and control, miR-216a inhibitors and control were synthesized in Ribobio (Shanghai, China). Then RT-qPCR and Western blot assays were performed to evaluate the transfection efficiency after 48 h.

### Western blot assay

Totally 10^5^ transfected PBMCs were collected and lysed with RIPA solution after 48 h post-transfection (Beyotime, Shanghai, China). The total protein was boiled after the concentration measurement with a BCA kit (Beyotime). Totally 15 μg of protein was separated with SDS-PAGE and transferred to PVDF membranes. Finally, the protein was visualized with Western-Ready ECL Substrate Kit (BioLegend, San Diego, CA, USA). The primary antibodies were used as anti-GAPDH (ab38168, Abcam), AMPK (ab32047, Abcam), p-AMPK (ab133448, Abcam), p38MAPK (8690, Cell Signaling) and p-p38 MAPK (4511, Cell Signaling).

### ELISA assay

The protein levels of IL-6, TNF-α, and IL-1β in cell culture medium were detected with corresponding Human Quantikine ELISA Kit (R&D Systems, Minneapolis, MN, USA). The experiments were performed according to the instructions.

### Cell viability analysis

Cell Counting Kit-8 (CCK-8, Beyotime, Shanghai, China) was used for cell viability analysis. Totally 3000 PBMCs were planted in 96-well plates and treated with 0.1 μM Doxorubicin for 24h or 48h as previous report [41]. We added 10 μl CCK-8 reagent into each well and incubated for 2 h at 37° C. Then cell viability was measured with the absorption at 450 nm with a microplate reader (Tecan, Switzerland).

### RNA immunoprecipitation (RIP) assays

RNA immunoprecipitation (RIP) assays were performed as previous report [42]. DNA probes for RNA interaction analysis were synthesized by Ribobio. EZ-Magna RIP Kit (Millipore, Billerica, MA, USA) was used for RIP assays. Transfected PBMCs were lysed with complete RNA lysis buffer, then added magnetic beads conjugated with human anti-Argonaute2 (Ago2) antibody (Millipore) and control IgG (Millipore). The RNAs contacted with immunoprecipitates were collected with TRIzol reagent. RT-qPCR assays were performed for the levels of miR-216a.

### Dual luciferase reporter analysis

PBMCs were transfected with wild type or mutant primGLO-p38MAPK-3’UTR. Then co-transfected with miRNA-216a and miR-NC, respectively. Luciferase activity was analyzed with Dual-Luciferase Reporter assay kit (Promega, Madison, WI, USA) after 48h of culture. Firefly luciferase activity was normalized to Renilla luciferase activity.

### Statistical analysis

Statistical analysis was performed with GraphPad Prism 8.0 (GraphPad Inc., San Diego, CA, USA). All experiments were independently conducted for at least 3 times. Receiver operating characteristic (ROC) analysis were performed for diagnostic values of lncMIAT. One-way ANOVA was used for the comparison among groups. Pearson correlation model was used for the correlation between lncMIAT and p38MAPK. P < 0.05 was considered as statistical difference.
